# Establishment of the fetal-maternal interface: developmental events in human implantation and placentation

**DOI:** 10.3389/fcell.2023.1200330

**Published:** 2023-05-17

**Authors:** Chien-Chu Huang, Ya-Wen Hsueh, Chia-Wei Chang, Hsi-Chen Hsu, Tung-Chuan Yang, Wu-Chou Lin, Hsun-Ming Chang

**Affiliations:** Department of Obstetrics and Gynecology, China Medical University Hospital, Taichung, Taiwan

**Keywords:** embryo implantation, trophoblast differentiation, human placenta, maternal immune tolerance, fetal-maternal cellular trafficking, placenta-enriched molecules

## Abstract

Early pregnancy is a complex and well-orchestrated differentiation process that involves all the cellular elements of the fetal-maternal interface. Aberrant trophoblast-decidual interactions can lead to miscarriage and disorders that occur later in pregnancy, including preeclampsia, intrauterine fetal growth restriction, and preterm labor. A great deal of research on the regulation of implantation and placentation has been performed in a wide range of species. However, there is significant species variation regarding trophoblast differentiation as well as decidual-specific gene expression and regulation. Most of the relevant information has been obtained from studies using mouse models. A comprehensive understanding of the physiology and pathology of human implantation and placentation has only recently been obtained because of emerging advanced technologies. With the derivation of human trophoblast stem cells, 3D-organoid cultures, and single-cell analyses of differentiated cells, cell type-specific transcript profiles and functions were generated, and each exhibited a unique signature. Additionally, through integrative transcriptomic information, researchers can uncover the cellular dysfunction of embryonic and placental cells in peri-implantation embryos and the early pathological placenta. In fact, the clinical utility of fetal-maternal cellular trafficking has been applied for the noninvasive prenatal diagnosis of aneuploidies and the prediction of pregnancy complications. Furthermore, recent studies have proposed a viable path toward the development of therapeutic strategies targeting placenta-enriched molecules for placental dysfunction and diseases.

## 1 Introduction

Achieving a successful pregnancy relies on a series of complex interactions between the fetus-derived placenta and maternally derived decidua and the molecular signals that trigger intrauterine programming (Fowden et al., 2008). These cellular interactions are coordinately regulated at multiple levels, from systematic endocrine hormones to direct contact with paracrine and juxtacrine factors between trophoblasts and decidual cells in the maternal endometrium (Xu et al., 2021). The placenta is a highly complex and transient endocrine organ that is critical in integrating maternal and fetal signals to control the selective exchange of gas, nutrients, and waste between maternal and fetal circulation (Jansson, 2016). Notably, the placenta secretes various hormones and specific factors that help create an appropriate intrauterine environment for fetal growth and development (Li et al., 2021a).

The placenta develops from the trophectoderm of the activated blastocyst in both humans and mice (Hamatani et al., 2004). Upon implantation, invasive trophoblasts anchor the blastocyst to the decidualized uterine epithelium, where placentation occurs. During this period, placental trophoblasts differentiate into different cell subtypes that extensively remodel the maternal decidual endometrium and uterine vessels. Cell-cell interactions result in the adaptation and maintenance of maternal-fetal immune tolerance, eventually establishing a unique environment at the fetal-maternal interface (Wu et al., 2022). Fetal development is underpinned by the placenta, with its functional or developmental defects that may compromise and predispose the fetus to a number of chronic adult diseases and psychiatric disorders in adulthood (Bronson and Bale, 2016; Burton et al., 2016).

Given the complexity of the placenta in maternal-fetal communication, the dynamic metabolism and diverse functions of the placenta at different developmental stages of normal pregnancy remain largely elusive. Additionally, various types of trophoblasts, including trophoblast stem cells, progenitor cells, and different subtypes of differentiated trophoblasts, and their individual roles in the physiological and pathophysiological conditions of placentation are not completely understood. Indeed, our knowledge of how inadequate or impaired placentation contributes to pregnancy-related complications is primarily based on the presumption that such placental dysfunction develops at the early stage of pregnancy, when it is difficult to predict which placental tissues would have developed pathological conditions during late gestation. Therefore, our understanding of the developmental information and function of the placenta is primarily obtained from studies performed using mouse models. In this regard, mouse knockout studies have identified several critical regulatory genes, some of which are detected in the human placenta (Simmons and Cross, 2005; Latos and Hemberger, 2014). Although both humans and mice show some similarities, such as hemochorial placentation, in which fetus-derived trophoblasts directly contact maternal blood, different implantation processes, trophoblast invasion, key regulators of trophoblast development, placental structure, and placental vascularization occur between the two species (for reviews, see (Knöfler et al., 2001; Carter, 2007)). Taken together, these findings indicate that studies performed using mouse models are not ideal for interpreting the functional aspects of human placenta. To better understand the molecular mechanisms of human implantation and early placentation and further advance therapeutic strategies for placental dysfunction and pregnancy diseases, the establishment of appropriate human study models is urgently needed. In this review, we summarize the current knowledge of human embryo implantation, trophoblast differentiation and invasion, and early placentation as well as their underlying mechanisms. Additionally, we focus on different cell lineages of human peri-implantation embryos and trophoblasts and their unique cell markers. Furthermore, we review the current literature on placentation- and pregnancy-related disorders and describe the development of novel strategies for diagnosing, treating, and preventing associated diseases.

## 2 Human peri-implantation embryo

**FIGURE 1:**
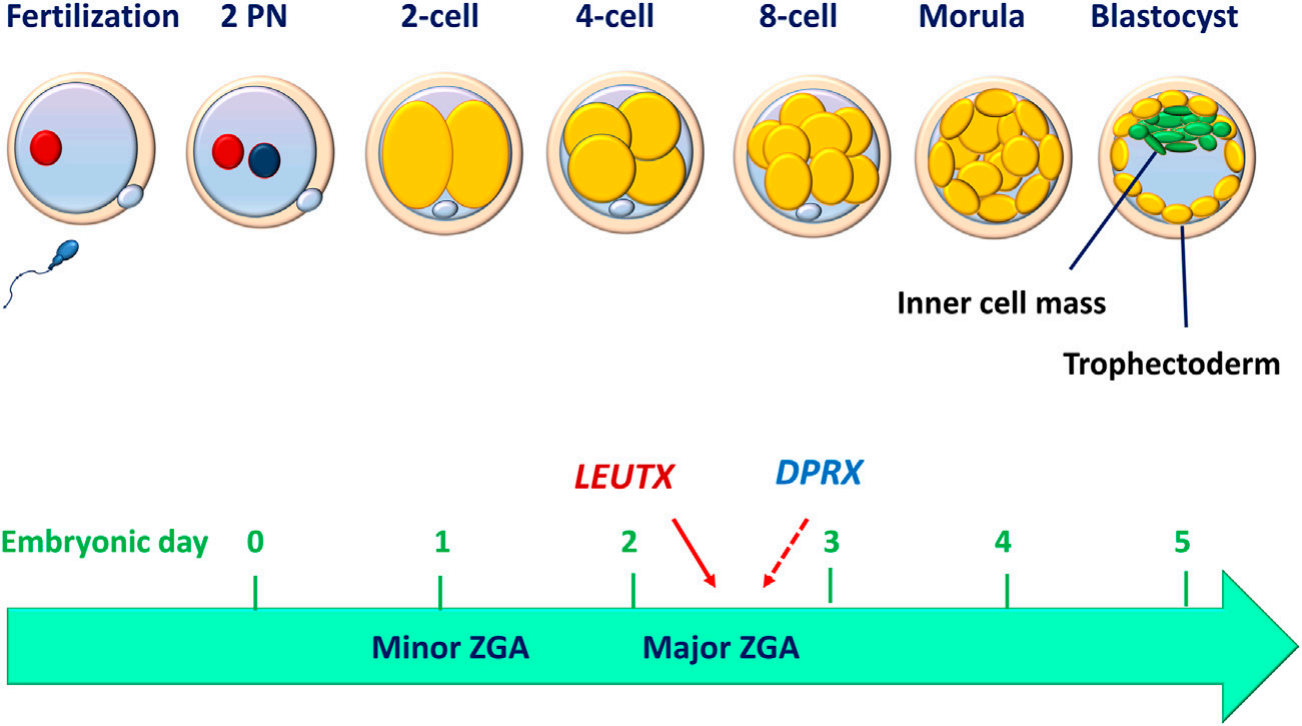
Human peri-implantation embryo development. Before implantation, human embryo undergo cell divisions culminating in the development of a blastocyst made up of a discernible inner cell mass and trophectoderm. The compaction of the embryo (morula) occurs between embryonic days 3 and 4, while the blastocyst formation occurs between days 5 and 6. The minor zygotic (embryo) genome activation (ZGA or EGA) begins as early as the 2-cell stage, while the major ZGA occurs at the 4- to 8-cell stage. LEUTX acts as a transcriptional activator at the 4-cell stage, whereas DPRX acts as a balancing repressor at the 8-cell stage during the process of ZGA.

Upon fertilization, the human oocyte resumes its second meiotic division with a rapid exchange of protamines in the paternal genome and histones in the maternal genome (Jansz and Torres-Padilla, 2019). These two haploid genomes decondense to become segregated pronuclei (Figure 1). The zygote and its associated membranes undergo five to six mitotic cell divisions (cleavages), leading to more cells without increasing the total volume of the embryo. After mitotic division, each daughter cell produced by cleavage is defined as a blastomere. Human embryo development begins in relative transcriptional silence with a maternal-to-zygote transition (MZT) and a pool of maternally transcribed mRNAs and proteins that induce zygotic (or embryonic) genome activation (ZGA or EGA) and the subsequent degradation of maternal transcripts (Lee et al., 2013). Studies performed in mammalian embryos using high-throughput methods have deepened our understanding of the molecular principles underlying MZT. In the peri-implantation embryo, mouse major ZGA occurs at the two-cell stage (26–29 h after fertilization), whereas human major ZGA begins at the four-to eight-cell stage (on day 3), although minor human ZGA occurs as early as the two-cell stage (for reviews, see (Niakan et al., 2012; Eckersley-Maslin et al., 2018)) (Figure 1). These chromatin remodeling events are critical in establishing the nuclear foundations essential for subsequent triggers of cell differentiation. An *in vitro* study performed using a human embryonic stem cell overexpression model demonstrated that *LEUTX* acts as a transcriptional activator at the 4-cell stage, whereas *DPRX* acts as a balancing repressor at the 8-cell stage during human ZGA (Jouhilahti et al., 2016) (Figure 1).

Following ZGA, the embryo subsequently becomes compacted to form a morphological feature with a radial symmetrical structure, the morula (Figure 1). The morula consists of blastomeres (embryonic cells) in a compact cluster that are contained within a glycoprotein membrane called the zona pellucida. Despite the lysis or fragmentation of one or more blastomeres, the human embryo continues to develop into a blastocyst that comprises a fluid-filled cavity (blastocoel) and an inner cell mass (ICM), which is surrounded by a group of cells that form the outer shell (trophectoderm, TE) (Figure 1). The human blastocyst develops at approximately 4–5 days after fertilization. The formation of TE represents the first lineage of the precursor for all trophoblast cells that segregates the ICM eventually giving rise to the embryo proper. At 6–7 days after fertilization, the interaction of the polar TE (the part of the TE that is adjacent to the ICM) with the uterine luminal epithelium (decidualized endometrium) leads to implantation, which is the first step in placental development. A recent single-cell RNA sequencing (scRNA-seq) study has shown that in both humans and mice, the transcriptomes of polar and mural TEs diverged after the embryos hatched from the zona pellucida, with polar TEs being more mature than mural TEs. (Liu et al., 2022). To achieve developmental competence, the peri-implantation embryo must undergo a highly orchestrated series of events that include fertilization, formation of pronuclei, syngamy, cell division (cleavage), ZGA, compaction, cell lineage differentiation and blastocyst formation (Zernicka-Goetz et al., 2009). During this period, environmental alterations, epigenetic modification, and embryonic metabolism may potentially affect the developmental competence of human embryos (Chason et al., 2011). However, *in vitro* studies performed using human embryos and human pluripotent stem cells indicate that the critical remodeling events at the peri-implantation stage of human development are embryo-autonomous or self-organizing in the absence of maternal tissues (Deglincerti et al., 2016; Shahbazi et al., 2016). Specifically, human blastocysts can self-organize to recapitulate many key features of *in vivo* embryo development (at least up to 12 days post-fertilization) after they are *in vitro* cultured in an attachment substrate without any maternal input (Deglincerti et al., 2016).

### 2.1 Cell lineages in the human peri-implantation embryo

**FIGURE 2:**
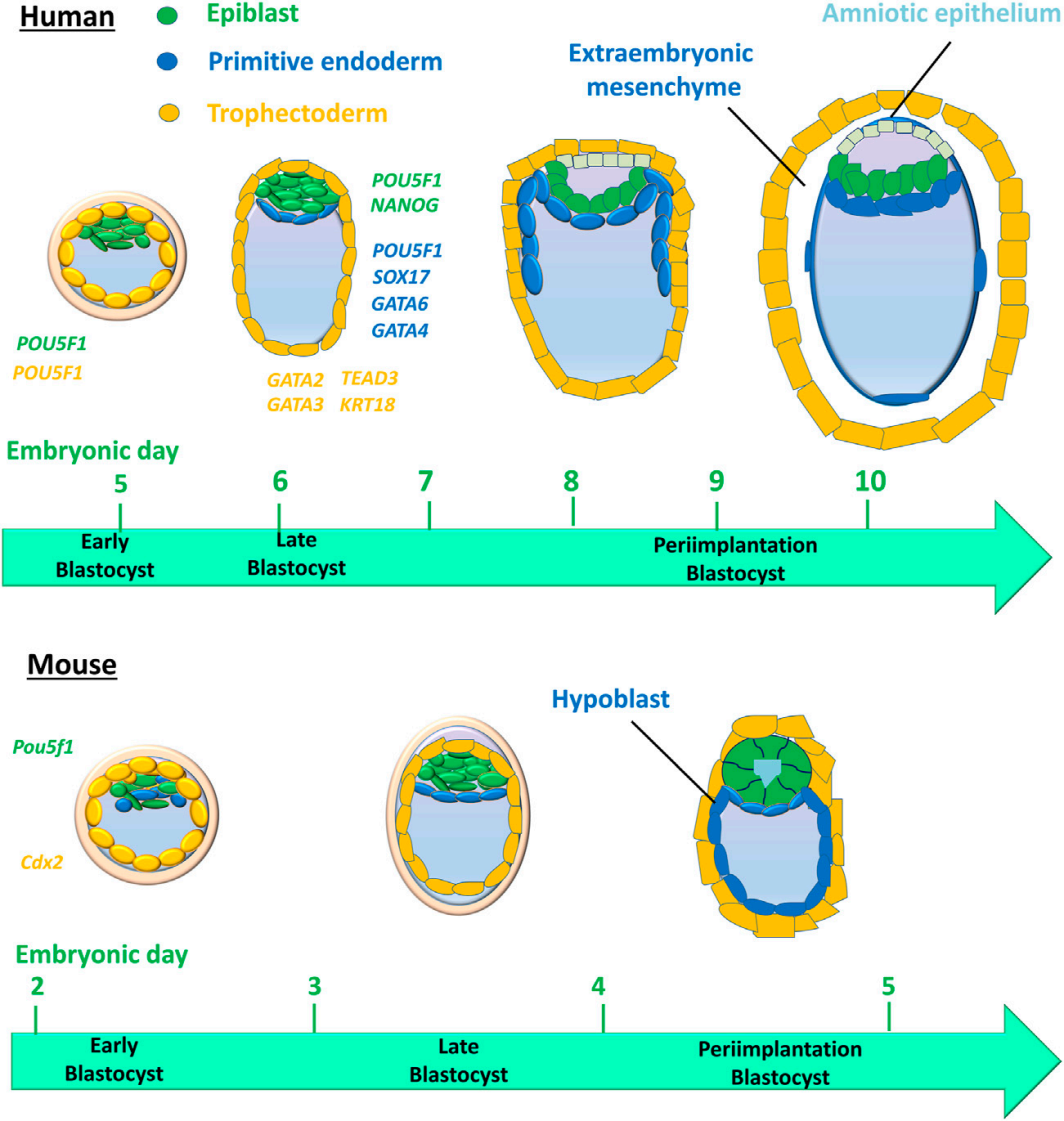
Cell lineages and their timing in humans *versus* mice during early embryo development. The first cell lineage segregation in the mouse embryo occurs at embryonic days (E) 2.75–3.25, critical timing during which the compact morula becomes an early blastocyst. Cells positioned inside the embryo are directed into the inner cell mass (ICM), whereas the cells positioned outside the embryo are directed into the first extraembryonic tissue, the trophectoderm that supports uterine implantation and the development of the placental epithelium. The second segregation further distinguishes two distinct ICM cell lineages: the second extraembryonic tissue, then the primitive endoderm (PE) that forms the primary yolk sac and pluripotent epiblast (EPI) that gives rise to the embryo proper. However, human embryos display a concurrent rather than stepwise cell lineage segregation, in which the PE, EPI, and TE emerge simultaneously. Colored italic text represents the cell expression of genes for cell lineages in human embryos.

Studies have shown that human and mouse cell lineage specification of embryos starts at the morula stage (Chazaud and Yamanaka, 2016; Sahakyan and Plath, 2016). Unlike in humans, cell fate decisions in mouse early embryo development are stepwise (Figure 2). The first cell lineage segregation in the mouse embryo occurs at embryonic day E) 2.75–3.25, a critical time when the compact morula (consisting of 10–30 cells) undergoes cavity formation to become the blastocyst. The cells positioned inside the embryo are directed into the ICM, whereas the cells positioned outside the embryo are directed into the first extraembryonic tissue, the TE that supports uterine implantation and development of the placental epithelium (Stirparo et al., 2018). The second segregation further distinguishes between two distinct ICM cell lineages: the second extraembryonic tissue, or primitive endoderm (PE), which forms the primary yolk sac, and the pluripotent epiblast (EPI), which gives rise to the embryo proper (Gardner, 1982; Stirparo et al., 2018). However, human embryos display concurrent rather than stepwise cell lineage segregation, in which PE, EPI, and TE emerge simultaneously (Chazaud and Yamanaka, 2016; Sahakyan and Plath, 2016) (Figure 2).

In the early mouse embryo, the pluripotency factor *Pou5f1* (also known as *Oct4*) is required for the formation of the ICM, and the caudal-like transcription factor *Cdx2* is required for the development of TE (Nichols et al., 1998; Strumpf et al., 2005) (Figure 2). However, in the early human blastocyst, *POU5F1* is expressed in both the TE and early ICM (Niakan and Eggan, 2013). The expression of *POU5F1* is downregulated in TEs by E6 in human blastocysts, but *POU5F1* remains at a high expression level in the ICM (Niakan and Eggan, 2013; Yan et al., 2013; Deglincerti et al., 2016). During the second lineage segregation (between E6 and E7), high levels of *POU5F1* and *NANOG* are co-expressed in the EPI, while a lower level of *POU5F1* as well as *SOX17*, *GATA6*, and *GATA4* are co-expressed in the PE (Kuijk et al., 2012; Roode et al., 2012; Niakan and Eggan, 2013; Deglincerti et al., 2016; Guo et al., 2016) (Figure 2). *GATA2*, *GATA3*, *TEAD3*, and *KRT18* are coexpressed in the TE (Yan et al., 2013; Blakeley et al., 2015; Deglincerti et al., 2016) (Figure 2). Cell-specific markers for 3 cell lineages (EPI, PE, and TE) in human embryos are listed in Table 1. A recent scRNA-seq study has shown that in both humans and mice, the transcriptomes of polar and mural TE diverged after the embryos hatched from the zona pellucida, with polar TE being more mature than mural TE (Liu et al., 2022).

**TABLE 1:** Cell-specific markers for 3 cell lineages (EPI, PE, and TE) in human embryos.

Trophectoderm (TE)	GATA2
	GATA3
	TEAD3
	KRT18
Epiblast (EPI)	NANOG
	SOX2
	TDGH1
	KLF17
Primitive endoderm (PE)	GATA4
	GATA6
	SOX17
	PDGERA

## 3 Embryo implantation

### 3.1 Implanting embryos

The implantation of the competent blastocyst into the receptive uterus is key for developing mammalian embryos. Successful implantation comprises the following three phases: apposition, attachment (or adhesion), and penetration. Blastocyst activation is defined as the programming of a blastocyst into an implantation-competent state (Paria et al., 1993). Embryo transfer studies performed using a delayed implantation mouse model have demonstrated that blastocyst activation is the major determining factor for implantation (Paria et al., 1993). Upon activation, the ICM of blastocysts is programmed for further development. As the first place that encounters an attachment reaction with the blastocyst, the luminal epithelium is a critical mediator that transmits molecular signals to achieve uterine receptivity. However, the stroma is another major player, given that studies performed using compartment-specific deletion mouse models have demonstrated that bidirectional communication between the stroma and epithelium is required for proper uterine receptivity and subsequent embryo implantation (Simon et al., 2009; Cha et al., 2012; Franco et al., 2012). To understand the molecular mechanisms underlying cell-cell communication between the TE and endometrium during implantation, we recently used an embryo–Ishikawa cell (which was established from an endometrial adenocarcinoma woman) coculture system to mimic *in utero* embryo implantation (Liu et al., 2022). Our recent scRNA-seq study demonstrated that embryos that fail to attach *in vitro* showed genetic aberrations (which were downregulated) in pathways related to protein metabolism, energy production, and 18 S ribosomal RNA m6A methylation (Liu et al., 2022). Similarly, using the preimplantation genetic testing (PGT) for genetic analysis before embryo transfer, one or 2 cells from the same biopsied cluster were isolated for transcriptome sequencing to identify key genes that might regulate embryo implantation. Our results showed that the translational elongation genes (such as *RPS28* and *RPS29*) were upregulated, while genes involved in protein metabolism, mitochondrion organization, and 18*S* rRNA m^6^A methylation as well as genes involved in implantation (*FGF13* and *RBP7*) and DNA repair (*C20orf196*, also known as *SHLD1*) were downregulated in cells from embryos that failed to implant during IVF/ET treatment (Liu et al., 2022).

Animal studies performed using rodent models have shown that the state of activity in the blastocyst is the major determining factor that initiates the implantation process in the receptive uterus (Carson et al., 2000; Kim and Fazleabas, 2004). In mice, embryonic diapause (or delayed implantation) is a naturally occurring phenomenon in which the dormant blastocyst exhibits most of the characteristics of the normal blastocyst, except for the metabolic activity that mediates implantation competence (Paria et al., 1993; Hamatani et al., 2004; Fu et al., 2014). Under the influence of ovarian hormones (estrogen and progesterone), the blastocyst is globally programmed to a competent state for implantation, a process defined as blastocyst activation (Paria et al., 1993). Embryonic diapause can be experimentally induced by performing an ovariectomy before E4 at the time of peri-implantation estrogen secretion, leading to a state of blastocyst dormancy (implantation incompetency) (Paria et al., 1993). Studies performed using this delayed implantation model have shown that the competent status of blastocysts can be rescued by administering estrogen (Paria et al., 1993). A study conducted using this delayed model showed that the inhibitory regulators of the mTOR and Myc signaling pathways as well as polyamine biogenesis induce cell cycle arrest and reduce the cellular metabolism that maintains blastocyst dormancy (He et al., 2019). Instead of polar TE, the mural TE differentiates into an invasive status that penetrates through the cellular tight junctions and extracellular matrices for further implantation (He et al., 2019). Additionally, the differential expression analysis revealed that the blastocyst acts as a proinflammatory item that triggers embryo-uterine attachment by secreting several proinflammatory substances, including TNFα and S100A9 (He et al., 2019). In both humans and mice, the implantation poles exhibit high transcriptional activity of *GATA3*, *RXRA*, *ARID3A*, and *BHLHE40*, indicating the important roles of these genes in embryo implantation (Liu et al., 2022).

### 3.2 Implantation window and markers for uterine receptivity

The menstrual cycle (approximately 28–30 days) begins with menses, followed by the follicular (proliferative) phase that is stimulated by the increasing estrogen secreted from developing follicles. During the follicular phase, estrogen induces the proliferation regeneration of endometrial tissues, including the epithelium, stroma, and vascular endometrium. At midcycle (on day 14), elevated estrogen exerts positive feedback on the surge secretion of pituitary gonadotropins (FSH and LH), which in turn triggers ovulation (Tomikawa et al., 2012). The early luteal (secretory) phase is characterized by the remarkable thickening of the endometrium accompanied by the formation of the corpus luteum (which is derived from the ruptured follicle). During this phase, the corpus luteum primarily secretes progesterone in preparation for embryo implantation. Stimulatory effects of progesterone lead to a series of endometrial ultrastructural changes, including secretory glands, endometrial edema, stroma cell differentiation, and an influx of leukocytes (a process called predecidualization) (Cha et al., 2012). In humans, embryo implantation occurs during the mid-luteal phase (days 20–24, day 4 in mice) at the time that increasing estrogen superimposed on progesterone accomplishes endometrial receptivity, a period defined as the implantation window (or window of receptivity) (Cha et al., 2012). Then, the endometrium proceeds to the nonreceptive (late luteal) phase until the next menstruation occurs (Wang and Dey, 2006). When successful implantation is achieved, the blastocyst secretes chorionic gonadotropin to support the corpus luteum and maintain pregnancy. Conversely, the mid-luteal phase spontaneously transitions to a refractory phase in the absence of a competent embryo, resulting in the event of luteolysis followed by menstruation, which resets the menstrual cycle (McCracken et al., 1999).

In most mammals, including humans and mice, estrogen and progesterone are the main regulators of a successful pregnancy, regulating uterine functions by coordinating multiple paracrine/autocrine factors in a spatiotemporal manner. Indeed, the functional receptors of these ovarian hormones (estrogen receptor *α* and progesterone receptor A, or ERα and PR-A) are expressed in all the major compartments of the uterus: the epithelium, stroma, and myometrium (Wang and Dey, 2006). Leukemia inhibitory factor (LIF) and Indian hedgehog (IHH) are crucial elements for implantation, and they act as downstream responsive genes for estrogen and progesterone, respectively (Hambartsoumian, 1998; Simon et al., 2009). LIF belongs to the interleukin-6 family of cytokines and plays an essential role in modulating uterine receptivity and implantation, because the targeted depletion of *Lif* in mice exhibits implantation failure (Stewart et al., 1992; Song et al., 2000). Data obtained from clinical studies showed that the endometrial expression of *LIF* is significantly higher at the time of implantation in fertile women than in infertile women (Laird et al., 1997; Hambartsoumian, 1998; Piccinni et al., 1998). IHH is primarily expressed in the epithelium and acts to promote stromal cell proliferation *via* interaction with its receptors (which are expressed in the stroma) (Matsumoto et al., 2002). In the mouse uterus, progesterone stimulates the expression of *Ihh*, which mediates epithelial-mesenchymal interactions essential for blastocyst implantation, because its targeted depletion in the uterus leads to poor uterine receptivity and implantation failure (Matsumoto et al., 2002; Lee et al., 2006). Similarly, progesterone upregulates the expression levels of IHH and its receptor in human endometria, indicating its critical role in human implantation (Wei et al., 2010). Other factors that are responsive to ovarian hormones include tumor suppressor protein p53, FKBP52, steroid receptor coactivator 2 (SRC-2, Ncoa2), chicken ovalbumin upstream promoter-transcription factor (COUP-TFII,mNr2f2), and Hand2 (for reviews, see (Cha et al., 2012)).

### 3.3 Preparation of endometrium for implantation

In the menstrual cycle, the human endometrium is a highly regenerative tissue in response to estrogen stimulation during the proliferative phase (Wang et al., 2020). The dynamic change of endometrial tissue to sex steroid hormones is a complex process that is controlled by the interactions of various cell types, including epithelial, stromal, endothelial, and immune cells in the endometrium (Critchley et al., 2020). During the secretory phase following ovulation, the human endometrium transforms into a narrow window of receptive status to accept the embryo, which is called the window of implantation (WOI) (Wilcox et al., 1999). The scRNA-seq studies showed that the human WOI opens rapidly with a discontinuous transcriptomic activation in the epithelial cells, and this event is accompanied with a widespread decidualization change in the stromal fibroblasts (Wang et al., 2020). Aberrations of transcriptome expression of genes related to specific signaling pathways (cell cycle, SEMA3, EGF, PTN, and TWEAK) in stromal cells or decreased numbers of macrophages and natural killer cells may cause thin endometria, leading to infertility, recurrent implantation failure, recurrent pregnancy loss, and placental abnormalities (Lai et al., 2022; Lv et al., 2022). Additionally, there is a marked reduction of total uNK cells in the shed endometrium obtained from women with endometriosis compared with that from the normal controls (Shih et al., 2022). Furthermore, there is a decreased number of IGFBP1+ decidualized subset of endometrial stromal cells in the endometrium of women with endometriosis, indicating that compromised decidualization of stromal cells in these affected women (Shih et al., 2022).

During the menstrual cycle, decidualization is featured by the differentiation of elongated, fibroblast-like mesenchymal cells in the uterine stroma into rounded, epithelioid-like cells (Okada et al., 2018). Primarily secreted by the decidual stromal cells, IGFBP1 and prolactin have been widely used as markers of decidualization *in vitro* (James-Allan et al., 2018)*.* When the blastocyst successfully implants, the serum progesterone remains at a high level, which preserves the decidua and remodels the basal endometrial layer (Brosens et al., 2002). Decidualization is essential for trophoblast invasion and placentation, because various knock-out mouse studies have shown that the targeted depletion of specific genes (*Hoxa10* or *Src*) related to decidualization results in impaired decidualization, failed implantation and infertility (Lim et al., 1999; Shimizu et al., 2005; Lim and Wang, 2010). During early pregnancy, the decidual stromal cells maintain decidualization in response to the stimulation from the elevated intracellular cAMP levels, sustained activation of the PKA signaling pathway and the actions of progesterone (James-Allan et al., 2018).

### 3.4 Dynamics of implantation

**FIGURE 3:**
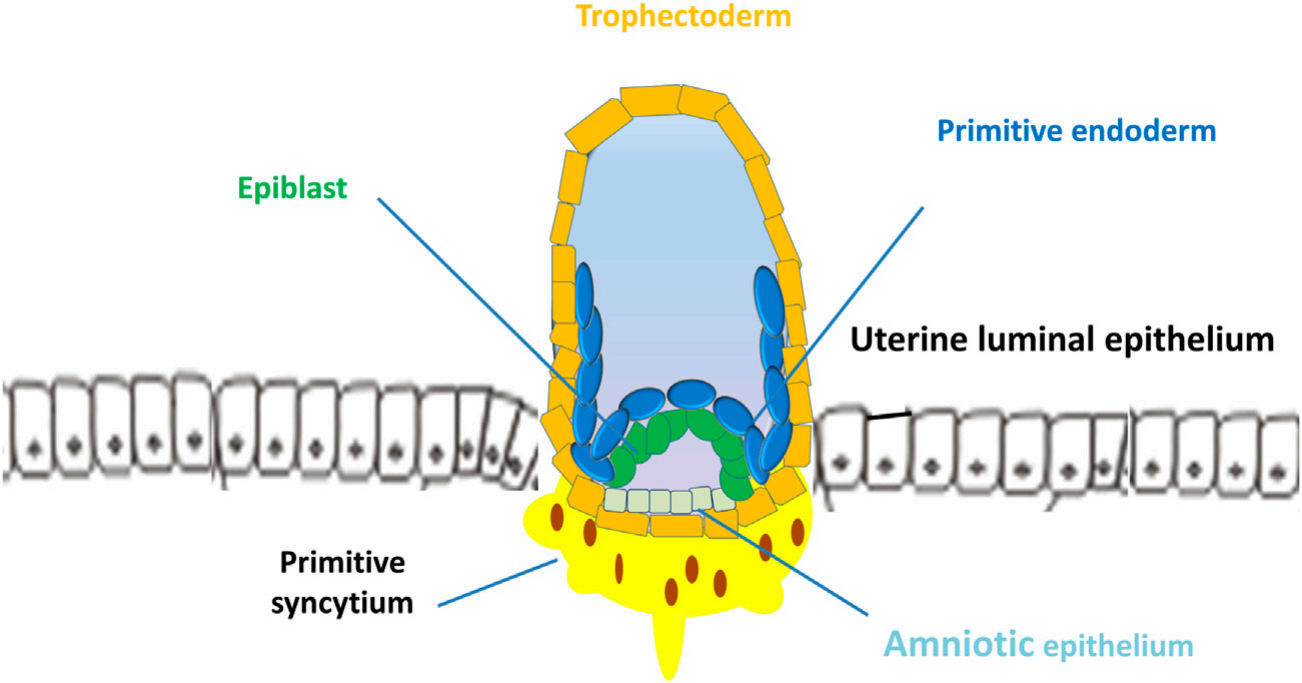
Implantation of human blastocyst into the uterine endometrium. The blastocyst is oriented with its inner cell mass toward the epithelium on days 6–7 when the first step of placental development commences. After implantation, the outer monolayer of the blastocyst, the trophectoderm, generates the first trophoblast lineages, which develop into 2 cell types, the early mononuclear cytotrophoblast and multinuclear primitive syncytium at day 8.

**FIGURE 4:**
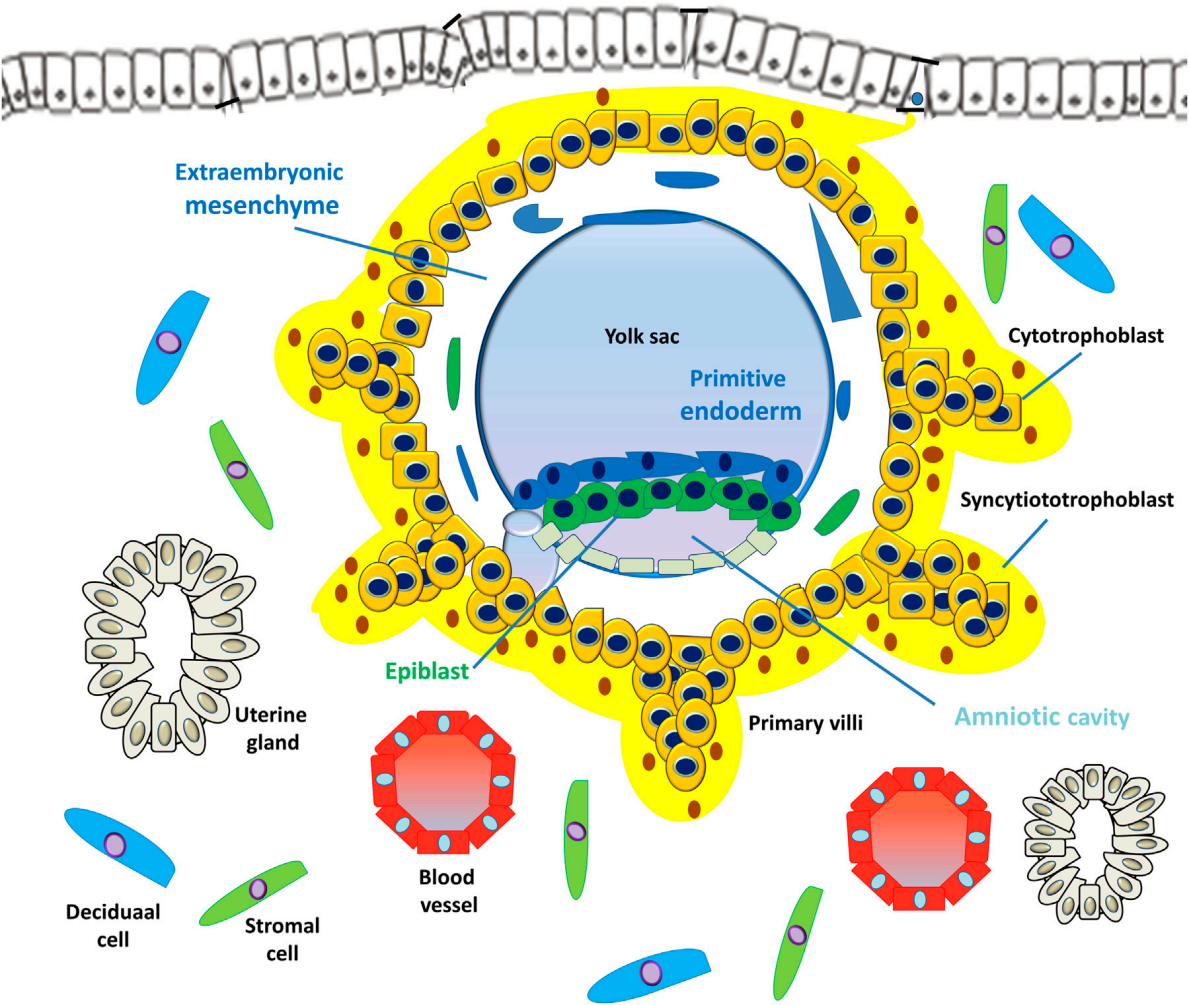
Development of embryonic disc and primary villi. The human embryo implantation process is intrusive; the blastocyst is embedded within the subepithelial stroma and further penetrates both the luminal epithelium and basal lamina. Upon primitive syncytium expansion, cytotrophoblasts proliferate and penetrate through the expanding syncytial mass to form the primary trophoblast villi (primary villi).

Uterine receptivity that matches blastocyst apposition is characterized by cellular and structural modifications of the endometrium: loss of epithelial cell polarity and formation of pinopodes (also known as uterodomes, apical epithelial cellular microprotrusions of the endometrium) (Nikas and Psychoyos, 1997; Blanco-Breindel et al., 2023). In mice, the implantation process starts with the placement of the blastocyst in a small tubular gland (crypt or nidus) because of the invagination of the luminal epithelium. Mouse trophoblasts further displace the luminal epithelium from the basal lamina and pass into the stroma of the endometrium. In humans, the implantation process is intrusive, and blastocysts are embedded within the subepithelial stroma (Figure 3). Human trophoblasts further penetrate both the luminal epithelium and basal lamina into the stroma of the endometrium (Figure 4) (Schlafke and Enders, 1975; Blanco-Breindel et al., 2023). During attachment, most mammalian blastocysts encounter increased vascular permeability at the apposition side of the endometrium. In mice, this phase occurs on E4 when the ICM of the blastocyst implants toward the lumen. In humans, the blastocyst is oriented with its ICM toward the epithelium on E6-E7 when the first step of placental development commences (Carson et al., 2000; Shibata et al., 2020) (Figure 3).

Following blastocyst attachment with the luminal epithelium, decidualization is initiated in the stroma bed where blastocysts implant. A functional luminal epithelium is required for the development of stromal decidualization, suggesting that specific factors and signals are transmitted by the epithelium to the stroma (Cha et al., 2012; Ochoa-Bernal and Fazleabas, 2020). This notion was confirmed by studies performed using conditionally deleted mouse models (knock out of *Msx1/Msx2* or *Klf5*) showing that mice with impaired epithelial function exhibit defective decidualization (Daikoku et al., 2011; Sun et al., 2012). In mice, the competent blastocyst is the major stimulus for the occurrence of decidualization, which is characterized by stromal cells that surround the blastocyst as it undergoes remarkable proliferation and differentiate into decidual cells with polyploidy. In human endometria, the initiation of predecidualization does not necessarily require the appearance of the blastocyst; however, the implanting blastocyst enhances the process (Cha et al., 2012; Ochoa-Bernal and Fazleabas, 2020). Taken together, these observations indicate that a functional network of interconnected compartments involving the luminal epithelium, stroma, and blastocyst is required to develop decidualization and uterine receptivity.

## 4 Trophoblast cell differentiation and invasion

After implantation, the outer monolayer of the blastocyst, the TE, generates the first trophoblast lineages, which develop into 2 cell types, the early mononuclear cytotrophoblast (CTB) and the multinuclear primitive syncytium (PS), at E8 (Boss et al., 2018) (Figure 3). Additionally, the ICM of the blastocyst develops into the second bilaminar extraembryonic tissue, the primitive endoderm (PE, also termed hypoblast, Hy), which in turn forms the primary yolk sac and pluripotent epiblast (EPI) that gives rise to the embryo proper (Gardner, 1982; Stirparo et al., 2018) (Figure 3). In primates, lineage tracing studies have shown that the PE also develops into the extraembryonic mesoderm (ExM), which subsequently forms the mesenchymal compartment of chorionic villi and the umbilical cord (Bianchi et al., 1993). However, evidence from molecular and lineage analysis studies shows that the ExM may be partially derived from the EPI, because the cells of these two lineages express several compatible markers (Sheng, 2015). At approximately E15, the EPI gives rise to the three embryonic germ layers and the amnion.

**FIGURE 5:**
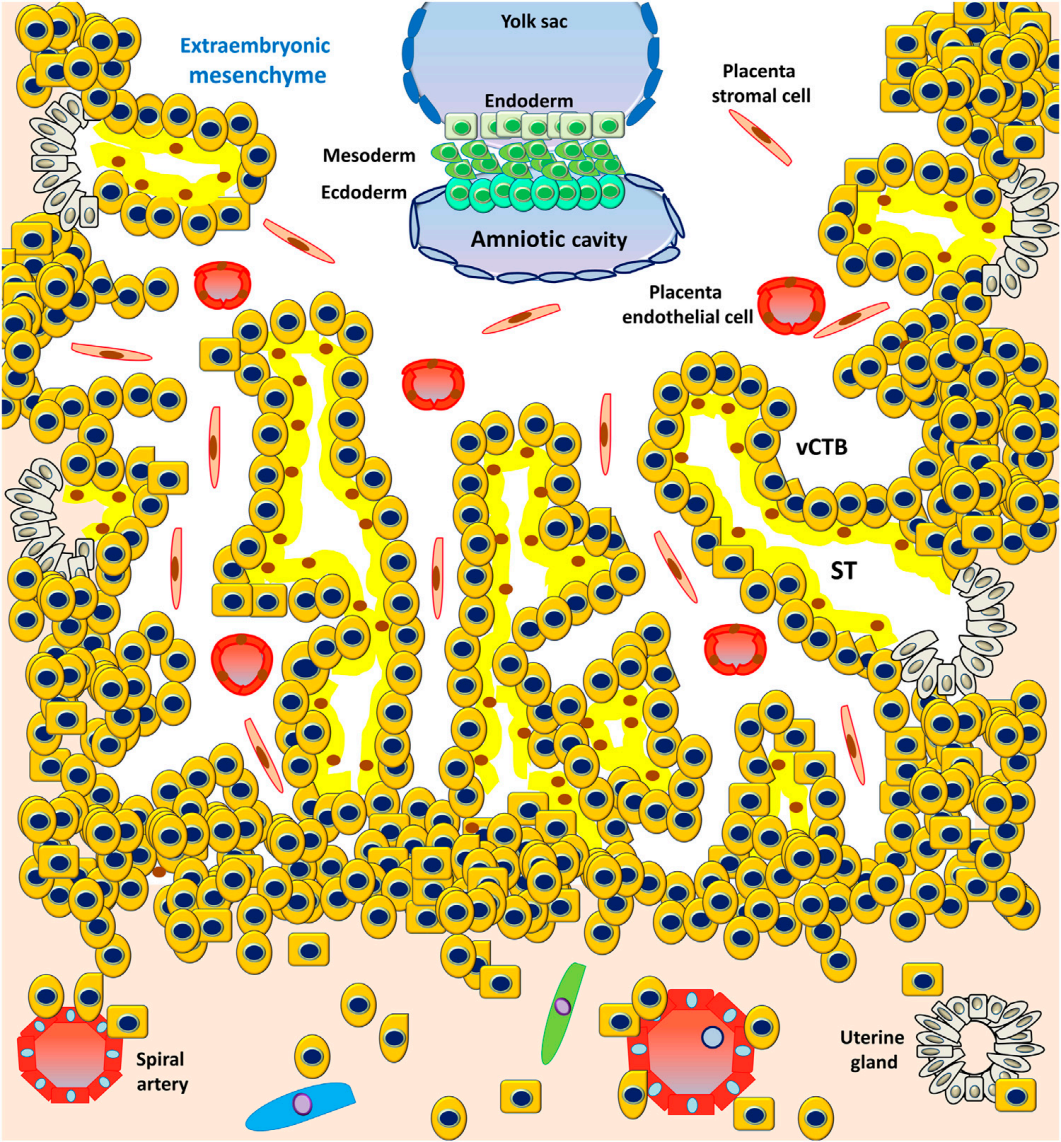
Development of the embryonic disc and tertiary villi. The primary villi extend through the underlying decidual endometria and erode into the uterine glands and blood vessels, followed by the migration of ExM cells into the structure of the primary villi, a transformation into the secondary villi. Proliferative CTBs associated with the secondary villi continue to tremendously expand, branch, and develop into villous cytotrophoblasts (vCTBs). The cell fusion of the developing vCTB generates an outer layer of multinuclear syncytiotrophoblasts (STs) that establishes an interface between the maternal blood and embryonic extracellular fluid, facilitating nutrient transport and gas exchange in the floating villi.

However, the PS is the first invasive trophoblast that expands into the decidual endometria (Figure 3). At E9, several vacuoles form in the PS, further fusing to form a dense network of lacunar spaces and subsequently breaching the uterine capillaries forming a discontinuous blood sinusoid (MS) at approximately E12-E13 (Hamilton and Boyd, 1960). The development of placental villi morphogenesis commences by E10. Upon PS expansion, CTBs proliferate and penetrate through the expanding syncytial mass to form primary trophoblast villi (primary villi) (Figure 4). The primary villi further extend through the underlying decidual endometria and erode into uterine glands and blood vessels. This process is followed by the migration of ExM cells into the structure of the primary villi, a transformation into the secondary villi during the following days. In addition, the proliferative CTBs associated with the secondary villi continue to tremendously expand, branch, and develop into villous cytotrophoblasts (vCTBs) (Figure 5). Cell fusion of the developing vCTB generates an outer layer of multinuclear syncytiotrophoblasts (STs) that establishes an interface between maternal blood and embryonic extracellular fluid, facilitating nutrient transport and gas exchange in floating villi (Figure 5). Arising from the asymmetrical cell division, fusion, and differentiation of vCTBs with the surrounding syncytium, the multinucleated STs form a superficial polyploid, a nonmitotic syncytial layer that secretes large quantities of placental hormones and growth factors to maintain the pregnancy, including progesterone, leptin, human chorionic gonadotropin (hCG), and human placental lactogen (HPL) (Evain-Brion and Malassine, 2003; Aplin, 2010).

At approximately E17-E19, the fetal circulatory system forms, and embryonic blood vessels enter the villi, forming the tertiary villi. At this moment, the fetal allantois (a hollow sac-like structure filled with clear fluid) extends, and three primary germ layers (the ectoderm, mesoderm, and endoderm) form. These villous blood vessels are derived from the ExM and eventually connect with the fetal vessels 4 weeks after conception (Burton et al., 2009a). Evidence from studies performed using cell lineages shows that the placenta leads the developmental process of *de novo* vascular formation, because the cells involved in early placental vasculogenesis and hemangiogenesis are derived from the ExM (Knöfler et al., 2019).

**FIGURE 6:**
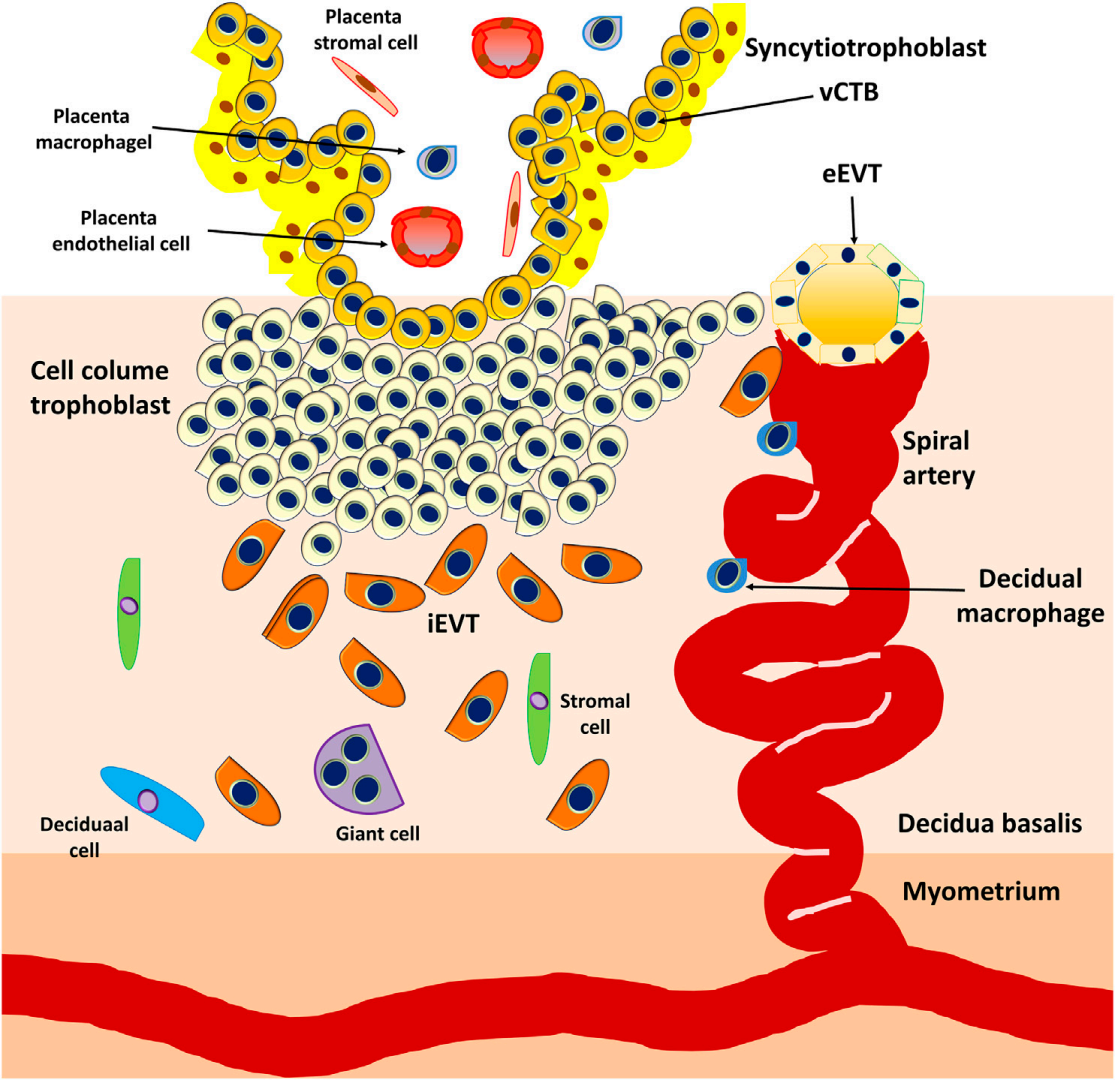
Development of anchoring villi, extracellular trophoblast cell lineage, and spiral artery remodeling. Extravillous trophoblasts are derived from the cytotrophoblasts located in the tips of the anchoring villi once mature villi (proliferative proximal cell column trophoblasts, pCCTs) have developed. The pCCTs serve as the progenitor cells of the differentiated EVTs. Two distinct EVT cell types (endovascular EVT, eEVT or eCTB and interstitial EVT, iEVT or iCTB) develop and the eEVTs remodel the uterine spiral arteries and promote maternal blood supply to the placenta, whereas the iEVTs invade the decidual stroma.

At approximately E15, the proliferating CTBs located at the distal sites expand laterally and form the outermost part of the placenta that envelops the conceptus, a structure called the trophoblastic shell. The trophoblastic shell firmly secures the placenta to the maternal endometrium called the decidua basalis, which is a critical anchorage between the placenta and the decidual endometria, acting to protect the embryo from oxidative stress (Burton and Jauniaux, 2017). In this shell, gaps between the villi and decidua basalis allow endometrial vessels to enter the intervillous spaces. During the early developmental process of placentation, cells of the trophoblastic shell differentiate into invasive extravillous trophoblasts (EVTs). However, EVTs can be derived from CTBs located in the tips of the anchoring villi once mature villi (proliferative proximal cell column trophoblasts, pCCTs) have developed (Figure 6). In this regard, pCCTs serve as the progenitor cells of differentiated EVTs (Knöfler et al., 2019). Placental EVTs invade through the decidual endometrium and further move toward the spiral arteries. These cells enter and facilitate the remodeling of arteries into large-bore, high conductance vessels that increase blood flow to the intervillous space (Burton et al., 2009b). At E15-E16, two distinct EVT cell types (endovascular EVT, eEVT, or eCTB and interstitial EVT, iEVT, or iCTB) have developed. The eEVTs remodel the uterine spiral arteries and promote maternal blood supply to the placenta, whereas the iEVTs invade the decidual stroma (Pijnenborg et al., 1980; Pijnenborg et al., 2006) (Figure 6). During early pregnancy, invasive iEVT provokes various actions to regulate placental function. In particular, iEVTs promote the immunological acceptance of the fetal allograft and control EVT function by interacting with neighboring cells, including decidual natural killer (dNK) cells, macrophages, and decidual stroma cells (Moffett et al., 2017; Pollheimer et al., 2018). Other EVT cell subtypes may exist, given that EVT cells have also been identified in endometrial glands, uterine veins, and lymphatics (Moser et al., 2010; Windsperger et al., 2017).

### 4.1 Cell lineages in trophoblast differentiation

All trophoblast cell lineages are derived from the TE cells of the blastocyst. The coordinated proliferation and differentiation of these cell lineages is critical to pregnancy establishment. Despite the development of different types of trophoblasts, including progenitors, stem cells, and differentiated subtypes, the pathophysiological roles of these cell types remain largely unknown (Knöfler et al., 2019). Our understanding of early placental development regulation is primarily based on the information obtained from studies conducted using mouse models. Animal studies using mouse trophoblast stem (TS) cells have provided useful information regarding the molecular and functional aspects of placental development (for reviews, see (Latos and Hemberger, 2014)). *In vitro* culture medium containing fibroblast growth factor 4 (FGF4), activin, and transforming growth factor-β1 (TGF-β1) maintains the self-renewal of mouse TS cells, which are able to differentiate into all trophoblast lineages (Tanaka et al., 1998). The maintenance of the undifferentiated state of mouse TS cells also involves several critical transcription factors, including *Cdx2*, *Elf5*, *Esrrb*, *Emos*, and *Gata3* (Latos and Hemberger, 2014). Given that humans and mice display similar hemochorial placentation, the two species display the direct contact of fetus-derived trophoblasts with maternal blood at the fetal-maternal interface. Despite their many similarities, significant differences have been observed in human and murine placentation in terms of structure and developmental events. Unlike in humans, mouse trophoblast invasion is relatively shallow, and factors derived from maternal sites determine the remodeling of uterine arteries (for reviews, see (Carter, 2007)). Additionally, the principal regulators that control human and murine placentation are different (for reviews, see (Knöfler et al., 2001)). Because of these discrepancies, studies using the mouse model are imperfect for investigating early placental development and related pregnancy complications in humans.

One of the most distinctive features of the human trophoblast is the expression profile of human leukocyte antigen (HLA) class I. The vCTBs fuse to form overlying multinuclear STs, and EVTs form placental bed giant cells that are located in the decidua endometrium and myometrium. Therefore, neither vCTBs nor STs express HLA class I, whereas EVTs express HLAs, including HLA-C, HLA-E, and HLA-G (a unique HLA expressed by trophoblasts) (Apps et al., 2009). In contrast to trophoblasts, most somatic cells express several HLAs, including HLA-A, HLA-B, HLA-C, and HLA-E antigens (Wei and Orr, 1990). In this regard, EVTs are the only human cells that uniquely express HLA-G but not HLA-A or HLA-B. Studies have shown that HLA-G is not coexpressed with either HLA-A or HLA-B in normal trophoblasts (Lee et al., 2016).

Traditionally, cytokeratin 7 (KRT7) and hCG are the most commonly used cell markers for determining trophoblasts (Lee et al., 2016). Some transcription factors, such as CDX2 and EOMES, which have been identified in the development of mouse TS cells, are considered markers for human trophoblasts. However, there are species differences between humans and mice in the network regulation and expression patterns during early placental development. KRT7 is a pan-trophoblast cell marker, whereas GATA3 and transcription factor activator protein-2 gamma (TFAP2C) are good cell markers for mononuclear trophoblasts but not multinuclear STs and villous stroma cells (Apps et al., 2011; Lee et al., 2016). Studies using an scRNA-seq analysis of human first-trimester placental cells have shown that all trophoblast cell lineages share the expression of KRT7 and PERP (p53 apoptosis effector related to PMP-22) (Suryawanshi et al., 2018). Additionally, these trophoblasts can be further subdivided into vCTBs, STs, and EVTs by expressing cell-specific markers, PARP1 (poly [ADP-ribose] polymerase 1), ERVFRD-1 (endogenous retrovirus group FRD member 1), and HLA-G, respectively (Lee et al., 2016; Suryawanshi et al., 2018). Cell-specific markers for human trophoblast cell lineages are listed in Table 2.

**TABLE 2:** Cell-specific markers for human trophoblasts.

All trophoblasts	KRT7
	PERP
Mononuclear trophoblasts	GATA3
	TFAP2C
Villous cytotrophoblasts	PARP1
Syncytiotrophoblasts	ERVFRD-1
Extravillous trophoblasts	HLA-G

In mice, ELF5 is a critical transcription factor that maintains self-renewal and thus the commitment to differentiate into the extraembryonic cell lineage in mouse TS cells (Donnison et al., 2005). Notably, the lineage-specific methylation of the Elf5 promoter is characterized in mouse embryos, hypermethylated in embryonic stem cells and hypomethylated in TS cells (Ng et al., 2008). Similarly, the promoter of ELF5 is mostly hypomethylated in early human placental tissue, indicating that the lack of methylation on the ELF5 promoter can be considered a cell marker for human trophoblasts (Hemberger et al., 2010).

Studies performed using microarray approaches show that human trophoblasts express specific noncoding microRNAs (miRNAs) (Bentwich et al., 2005). The chromosome 19 miRNA cluster (C19MC) is one of the largest miRNA clusters identified in the human genome, and it accommodates 46 miRNAs at the q arm of chromosome 19 (chr19q13.42) (Bortolin-Cavaillé et al., 2009). In cancer biology, C19MC miRNAs have been implicated in various invasive cancer cells (Augello et al., 2012). Notably, the expression of C19MC specifically marks human trophoblasts and embryonic stem cells (Bentwich et al., 2005; Noguer-Dance et al., 2010). The C19MC is a primate-specific miRNA that is maternally imprinted (Noguer-Dance et al., 2010). In humans, C19MC is highly expressed in human trophoblasts, indicating another potential candidate for defining human trophoblasts (Bortolin-Cavaillé et al., 2009; Donker et al., 2012).

### 4.2 Derivation of human trophoblast stem (TS) cells and establishment of the CTB 3D organoid culture system

TS cell populations have been isolated and developed from peri-implantation blastocysts in various mammalian species, including mice (Fléchon et al., 1995; Hashizume et al., 2006; Vandevoort et al., 2007). However, there is a significant difference in the regulation of lineage-associated placental development between species (Blakeley et al., 2015; Petropoulos et al., 2016). Although a great deal of information has been obtained from animal TS cells, understanding the development of human TS cells has been very challenging because of the distinct structural difference between human and animal placenta. Furthermore, the growth factors used to maintain animal TS cell self-renewal or culture conditions used to propagate these TS cells were unable to achieve human TS cells until recently (Kunath et al., 2014). The first human TS cells were isolated from first-trimester villi (Okae et al., 2018). Using an *in vitro* culture system consisting of the activation of Wingless/Integrated (Wnt) and the epidermal growth factor and inhibition of TGF-β, histone deacetylase (HDAC), and Rho-associated protein kinase (ROCK), the vCTBs can remain in an undifferentiated state for far longer than had previously been reported (Okae et al., 2018). Similar to the corresponding primary trophoblast cells, these human TS cells have the capacity to give rise to the three major trophoblast lineages (Okae et al., 2018). Notably, scRNA-seq analyses of these TS cells show transcriptomes similar to those of primary trophoblast cells (Okae et al., 2018). Intriguingly, lineage-specific markers associated with mouse TS cells are not predominantly expressed in human TS cells, indicating that these two species may have differential transcription networks for modulating trophoblast development.

Human CTB 3D-organoid (CTB-ORG) cultures have recently been generated from purified first-trimester CTB preparations (Haider et al., 2018; Cui et al., 2023; Dietrich et al., 2023). The established 3D organoids, which resembles the human placenta’s original structure and physiology, can maintain long-term self-renewal and expansion under specific culture conditions (Haider et al., 2018; Dietrich et al., 2023). Using global gene expression analyses, the CTB-ORGs were identified to express cell markers of trophoblast proliferation and stemness very similar to those in primary CTBs (Haider et al., 2018). Most importantly, the removal of growth factors required for self-renewal leads to cell outgrowth and differentiation into EVTs (expressing the NOTCH1 progenitor marker) and the formation of adjacent HLA-G^+^ EVTs (Haider et al., 2018). Additionally, the generation of a 3D organoid culture system using naive human pluripotent stem cells may help develop a placental environment and its susceptibility to emerging pathogens (Karvas et al., 2022). These studies have provided useful *in vitro* experimental systems for studying the sequential molecular steps of trophoblast cell column formation and differentiation.

### 4.3 Trophoblast cell invasion

During embryo implantation, EVT differentiation and invasion are critical for developing the human placenta and successful pregnancy outcomes. Inadequate placentation is characterized by defects in trophoblast differentiation or restricted EVT invasion and spiral artery remodeling, and it is associated with infertility, trophoblast cancers, and pregnancy-related complications, including preeclampsia, miscarriage, and fetal growth restriction (Wallace et al., 2012). At the fetal-maternal interface, human EVT invasion is stringently regulated by multiple factors, including growth factors, various adhesion molecules, and extracellular matrix components in an autocrine/paracrine manner (Graham and Lala, 1991; Chakraborty et al., 2002). Among these factors, recent studies have shown that TGF-β superfamily members, including TGF-βs, activins, inhibins, growth differentiation factors (GDFs), and bone morphogenetic proteins (BMPs), are multifunctional cytokines that regulate various cellular activities in EVTs (Cheng et al., 2013; Li et al., 2014; Li et al., 2015a; Li et al., 2015b; Cheng et al., 2015; Cheng et al., 2017; Zhao et al., 2018a; Zhao et al., 2018b; Cheng et al., 2018; Zhao et al., 2018c; Luo et al., 2020; Xie et al., 2020; Zhao et al., 2020; Li et al., 2021a; Luo et al., 2021; Yi et al., 2021; You et al., 2021; Luo et al., 2023). Emerging evidence reveals that TGF-β superfamily members and their putative receptors are expressed at the fetal-maternal interface (Wijayarathna and de Kretser, 2016; Li et al., 2021b; Haider et al., 2022). Through cellular and molecular genetic approaches, these cytokines have recently been shown to be closely involved in human embryo implantation and early placentation at the fetal-maternal interface (Ni and Li, 2017). Additionally, studies using human biological materials revealed that TGF-β superfamily members are essential for regulating human trophoblast differentiation toward invasive pathways, including interstitial EVT invasion and endovascular EVT invasion routes (Wijayarathna and de Kretser, 2016; Li et al., 2021b). With emerging technologies, including tissue microarrays, the clinical availability of recombinant human proteins, pharmaceutical development, new experimental settings, immortalized human cell lines, and advanced single-cell transcriptomics, functional studies have revealed divergent roles for TGF-β superfamily members in regulating human EVT invasion. Specifically, *in vitro* functional studies have shown that three activin isoforms (activin A, activin B, and activin AB) and BMP2 promote human EVT cell invasion, whereas TGF-β inhibits EVT cell invasion by modulating various cellular components, including matrix metalloproteinases, endothelial-like tube formation, connexins, cadherins, and cyclooxygenases (Cheng et al., 2013; Li et al., 2014; Li et al., 2015a; Li et al., 2015b; Cheng et al., 2015; Cheng et al., 2017; Zhao et al., 2018b; Zhao et al., 2018c). Furthermore, the dysregulation or variations in the levels of these ligands, their receptors, or related signaling pathways may affect their divergent effects on EVT invasion, leading to infertility or pregnancy-related complications (Ni and Li, 2017; Yi et al., 2021; You et al., 2021).

## 5 Early placentation

Although rodent models are frequently applied to study fetal-maternal interface establishment, they display substantial differences in placental structure, gestational period, and mechanisms of placentation compared with humans. In humans, the definite architecture of the placenta is established by the end of the third week after conception. Structurally, the human placenta comprises complex villous trees containing both anchoring and floating villi. The placental villous trees are surfaced by a single layer of contiguous multinuclear STs acting as the principal cellular barrier that separates the fetus from maternal blood. The underlying subjacent layer is the undifferentiated, mononucleated progenitor CTBs that can divide and fuse to replenish the STs. The CTBs at the tips of the anchoring villi can differentiate into mononucleated EVTs. Based on histological classification, the human placenta is defined as hemochorial because the placental villi are in direct contact with the maternal blood that fills the intervillous space (Soares et al., 2018). During the first trimester, the human placenta is defined by its hemodichorial pattern, a placenta with a double trophoblastic layer (the CTBs and STs) (Furukawa et al., 2014). With advancing gestation, the human placenta grows and becomes hemomonochorial with only one layer of STs because the underlying CTB layer becomes dispersed, thin, and discontinuous during the second and third trimesters. Aside from functioning as the primary endocrine cells of the placenta, STs act to facilitate nutrient, gas, and waste transport across the fetal-maternal interface.

EVTs are the main trophoblasts that anchor the human placenta to the decidual endometrium. The migration of EVTs to remodel the spiral arteries of the first third of the myometrium represents the other principal process of human placentation (Figure 6). During early pregnancy, in combination with dNK cells and macrophages, iEVTs (or iCTBs, a subtype of EVTs) migrate into the spiral arteries, where these cells initiate the remodeling process (Smith et al., 2009; Wallace et al., 2012). The iEVTs then differentiate into eEVTs (or eCTBs, a vascular adhesion phenotype of EVTs) that further interdigitate into the endothelial layer of the vessels, where eEVTs replace endothelial cells by inducing cell apoptosis (Zhou et al., 1997). The remodeling process dramatically changes the narrow spiral arteries into dilated vessels with high conductivity, ensuring maximal perfusion at the fetal-maternal interface.

In addition to remodeling the spiral arteries, eEVTs can form trophoblast during early gestation plugs to occlude the spiral arteries in the decidua basalis underlying the embryo implantation site. This vessel occlusion thus creates a low-oxygen environment that protects the fetal-placental unit from oxidative damage and promotes early placental development, angiogenesis, and vasculogenesis (Knöfler et al., 2019). Indeed, incomplete trophoblast plugging of the spiral arteries leading to a premature increase in oxygen concentration is reportedly associated with miscarriage (Khong et al., 1987; Hustin et al., 1990). At the end of the first trimester, the trophoblast plug is progressively eroded and accompanied by a significant increase in oxygenated maternal blood flow into the intervillous space (Roberts et al., 2017). With the disintegration of spiral artery plugs, the high oxygenated blood flow initiates the degeneration of the trophoblast layer, leading to the regression of placental villi and the formation of a mature form of the placenta (a discoidal shape) later in gestation (Knöfler et al., 2019). Given that there is no direct contact of the human placenta with maternal blood until the end of the first trimester, we may use this physiological event to distinguish between two gestational stages of pregnancy, early (first trimester) and late (second and third trimesters) stages.

Upon the establishment of placentation during the first trimester, the intervillous space is filled with fluid that contains substantial substances secreted by the uterine glands. Studies have shown that the uterine glands provide histotrophic nutrition for the developing fetus by delivering secretions into the placental intervillous space (Burton et al., 2002). For instance, STs phagocytose maternal uterine gland-secreted glycoproteins, such as mucin MUC-1 and glycodelin A, for dominant nutrient support (Burton et al., 2002). Thus, uterine glands are an essential source of fetal nutrients during early pregnancy, when the metabolic environment is essentially anaerobic. In addition to the principal nutrition source, uterine glands modulate placental growth and development by secreting a number of growth factors, including TGF-β, epidermal growth factor, vascular endothelial growth factor, and LIF (Hempstock et al., 2004).

## 6 Maternal immune responses and tolerance at the fetal-maternal interface

Normal pregnancy is a process of physiological stress that requires a delicate balance between the effects of proinflammatory and anti-inflammatory factors. Adaptation failure or the disturbance of this balance during embryo implantation and placentation has been associated with implantation failure and pregnancy-related complications (Kheshtchin et al., 2010; Szarka et al., 2010). The decidual endometrium is replete with activated immune cells; therefore, the successful implantation of the foreign allogenic embryo in the pregnant uterus highly relies on the establishment and maintenance of maternal-fetal immune tolerance.

### 6.1 Maternal immune responses during pregnancy

In addition to stromal cells, the decidual endometrium consists of a substantial portion (approximately 40%) of maternal leukocytes. During early pregnancy, dNK cells are the major (approximately 70%) immune cells, followed by decidual macrophages (20%–25%) and T cells (3%–10%) at the site of implantation [for reviews, see (Liu et al., 2017)]. These decidual leukocytes are recruited by decidual stroma and trophoblast cells in a chemokine gradient manner, and their phenotypes and functional characteristics are distinct from those in the maternal peripheral circulation (Huang et al., 2008; Zhao et al., 2011). With regard to angiogenesis during placentation, there are remarkable similarities between the invading trophoblast cells and cancer cells. Similar to cancer cells in oncogenesis, the underlying mechanisms by which decidual immune cells (especially dNK and regulatory T cells) promote angiogenesis are mediated by the secretion of various chemokines, cytokines, and angiogenic factors (Zhang et al., 2012).

A maternal active inflammation-like response starts from exposure to the seminal antigen at coitus (Sharma, 2014). In fact, several cytokines, including LIF, granulocyte colony-stimulating factor (G-CSF), interleukin (IL)-1, IL-6, and IL-11, have been shown to play pivotal roles in regulating decidualization and implantation (Stewart et al., 1992; Singh et al., 2011). In particular, seminal fluid-derived cytokines and chemokines attract regulatory T (Treg) cells to the endometrium (Schjenken et al., 2016). Additionally, maternal dendritic cells recognize the cross-presented seminal fluid and fetal antigens and transform effector CD4^+^T cells into Treg cells, which are then recruited to the endometrium (Sharma, 2014). Notably, the beneficial inflammatory response in initiating embryo implantation has been supported by the unexpected beneficial effect of endometrial biopsy-induced injury on the implantation outcome in patients undergoing *in vitro* fertilization (IVF) (Barash et al., 2003; Gnainsky et al., 2010). Taken together, these studies suggest that an inflammatory response and microenvironment are required to enhance uterine receptivity for embryo implantation.

### 6.2 Immune tolerance at the fetal-maternal interface

A successful pregnancy is based on the establishment of an immune tolerance that permits the uterus to carry the genetically different (allogenic) fetus while maintaining the maternal immune competence. Maternal immune tolerance at the fetal-maternal interface is achieved through multiple overlapping innate and adaptive immune mechanisms mediated by the restriction and modulation of decidual leukocytes and trophoblast cells (Solano, 2019). An abundance of dNK cells gain access to the fetal-maternal interface. These cells interact with decidual stromal and trophoblast cells, leading to an alteration in the functional profile and regulatory phenotype of decidual leukocytes (Nancy et al., 2012; Ander et al., 2019). During the first trimester, explants from human placental tissues produce factors, including G-CSF, TGF-β, and IL-10, which promote the differentiation of two types of cells in peripheral circulation: monocytes into M2MØ cells and T cells into Treg cells (Svensson-Arvelund et al., 2015). The polarity of these transformed cells most likely contributes to a homeostatic and tolerant immune microenvironment essential for stable fetal development (Svensson-Arvelund et al., 2015). Studies performed using animal and human models show that the number of Treg cells is markedly decreased during miscarriage (Alijotas-Reig et al., 2014). To create a tolerant microenvironment at the fetal-maternal interface, Treg cells suppress fetal allorejection by upregulating the expression of a number of immune modulatory molecules, including TGF-β1, IL-10, and heme oxygenase 1 (HO-1) and downregulating the expression of Th1 cytokines (cytokines responsible for causing macrophages to attack organisms and infected cells) (Zenclussen et al., 2002; Choi et al., 2005). *In vivo* studies have shown that Treg cell sensitization induced by paternal antigens is required for maternal immune tolerance (La Rocca et al., 2014).

Apoptosis is another mechanism applied to mediate the creation of maternal immune tolerance and the immune privilege of the fetus (Clark, 2005; Stenqvist et al., 2013). In particular, human STs secrete exosomes that express two bioactive surface molecules, the Fas ligand and the TNF-related apoptosis-inducing ligand (Stenqvist et al., 2013). These molecules further bind to their cognate death receptors, which are located on the decidual leukocytes that convey apoptosis, also suggesting the exosome-mediated immune privilege of the fetus (Stenqvist et al., 2013).

As a member of the nonclassical major histocompatibility complex (MHC), the HLA-G leukocyte antigen is a critical immunomodulatory molecule for embryo implantation and the establishment of immune tolerance (Ferreira et al., 2017). Uniquely expressed in human EVTs, HLA-G protects trophoblast cells from dNK cell-mediated cell lysis by binding to two dNK inhibitory receptors, LILRB and KIR2DL4 (Rajagopalan and Long, 1999; Apps et al., 2007). At present, many fundamental questions regarding the detailed molecular mechanisms by which HLA-G modulates fetal-maternal immune tolerance are only starting to be elucidated. In this regard, murine models are not suitable for investigating HLA-G-related function, because there is no consensus HLA-G orthologous gene in mice (Ferreira et al., 2017). However, the counterpart molecule of HLA-G in mice, a complement regulator, Crry, is the key immunomodulatory molecule that protects murine fetuses from complement-mediated cytotoxicity (Xu et al., 2000). Because Crry is murine specific, whether human trophoblasts or decidual cells express similar complement regulators to suppress complement activation and deposition remains to be determined.

Increasing evidence has suggested that miRNAs are also involved in the maintenance of maternal immune tolerance (Kamity et al., 2019). In the human placenta, miRNAs are present in the extracellular fluid and packed within placental cell-secreted extracellular vesicles. As early as the sixth week of gestation, the human placenta is an abundant source of extracellular vesicles (Mitchell et al., 2015). Specific miRNAs contained within placental extracellular vesicles have recently been proposed to mediate the tolerance phenotype at the fetal-maternal interface induced by repeated exposure to implantation- and placentation-induced inflammatory responses (Kamity et al., 2019). Additionally, human blastocysts express miRNA, which is essential for successful implantation and subsequent embryo survival (Kamity et al., 2019). Various miRNAs have been reported to tolerate the maternal immune system by regulating the function and differentiation of several innate immune cells (Nahid et al., 2011; Kumar Kingsley and Vishnu Bhat, 2017). For instance, miR-146 is an inhibitor targeted to the Toll-like receptor (TLR) signaling pathway of the innate immune response in decidual leukocytes, which plays a principal role in mediating maternal immune tolerance (Taganov et al., 2006; Vergadi et al., 2018). It was concluded that failed acquired immunity or impaired immune tolerance at the fetal-maternal interface will lead to a pathological response responsible for various adverse early and late pregnancy outcomes.

## 7 Human placental pathology and diseases

### 7.1 Recurrent pregnancy loss

Recurrent pregnancy loss is defined as women who have had two or more consecutive clinical pregnancies until 20 (defined by the ASRM) or 24 (defined by the ESHRE) weeks of gestation (Medicine, 2012). Approximately 5% of reproductive women suffer from two consecutive pregnancy losses, and 75% of failed pregnancies are due to implantation failure (Norwitz et al., 2001). Studies using various animal models have demonstrated that a defective implantation process can create detrimental effects that result in poor pregnancy outcomes (Cha et al., 2012). In humans, the window of uterine receptivity is crucial for successful conception, and any implantation beyond this window leads to spontaneous abortion (Wilcox et al., 1999). Multiple risk factors have been proposed for recurrent implantation failure, including advanced maternal age, smoking, elevated body mass index, stress, endocrine disorders, and embryonic abnormalities (e.g., aneuploidy) [for reviews, see (Bashiri et al., 2018; Ma et al., 2022)]. Uterine pathologies (polyps, myomas, and congenital uterine anomalies) and paternal effects on human embryo development (sperm DNA fragmentation) should be taken into consideration (Tesarik et al., 2004; Saravelos et al., 2008).

Immunological factors, specific autoantibodies, and infectious organisms causing chronic endometritis should be evaluated in women with recurrent implantation failure (Bashiri et al., 2018). In general, an increased number of Th1 cells is associated with the rejection of the embryo, whereas an increased number of Th2 cells is associated with the implantation of the embryo, and these cells are quantified by measuring their secreted cytokines (Nakagawa et al., 2015). Specifically, cytokines (such as TNF-α) produced by Th1 cells inhibit trophoblast growth and implantation, whereas cytokines (IL-4, IL-6, and IL-10) produced by Th2 cells suppress Th1 cell-induced tissue factor by monocytes (Robertson et al., 2018). Clinical studies show that the mean TNF-α/IL-4 ratio is significantly higher in women with multiple implantation failures than in normal controls (Kwak-Kim et al., 2003). LIF is a key factor linked to endometrial receptivity. In the clinic, this immune molecule is considered a possible cause of unexplained infertility, because lower LIF levels have been associated with a higher risk of multiple implantation failures (Hambartsoumian, 1998; Seli et al., 2005).

Other molecules involved in the implantation process include prostaglandins and cellular adhesion molecules. Studies have shown that decreased prostaglandin synthesis in the endometrium is associated with repeated implantation failure during IVF (Achache et al., 2010). Integrins, especially α1β1, α4β1, and αvβ3, are the major types of cellular adhesion molecules that function in cell-cell interactions during the implantation window and play a pivotal role in implantation, because lower expression levels of these integrins are associated with delayed histological development or an out of phase endometrium that decrease the implantation rate (Thomas et al., 2003).

### 7.2 Ectopic pregnancy

Ectopic pregnancy is defined as embryo implantation outside the uterine endometrium, with approximately 98% occurring in the fallopian tube (tubal pregnancy) (Khan et al., 2006; Shaw et al., 2010). Multiple etiologies have contributed to the occurrence of ectopic pregnancy, including pelvic inflammatory disease, advanced maternal age, smoking, and IVF procedure [for reviews, see (Shaw et al., 2010)]. Notably, the incidence of tubal pregnancy following the IVF procedures (approximately 4%–6%) increases two-to threefold compared to that of spontaneous pregnancy (approximately 1%–2%) (Farquhar, 2005). The possible contributing factors that cause a higher incidence of ectopic pregnancy in women undergoing IVF include the technique used for embryo transfer, having a thin endometrium, the ovulation stimulation protocol, and a higher estrogen concentration during the assisted reproduction cycle (Shao et al., 2012; Muller et al., 2016; Ma et al., 2017). Ectopic pregnancy occurs more frequently during induction protocols using gonadotropin-releasing hormone (GnRH) antagonists than those using a GnRH agonist flare-up and luteal GnRH agonist, indicating an extrapituitary role for GnRH in regulating the uterine and tubal environment during IVF treatment (Londra et al., 2016; Peng et al., 2016). Studies of functional ER subtypes using dual immunofluorescence analysis suggest that ERα and ERβ are coexpressed in ciliated and secretory epithelial cells and smooth muscles of the human fallopian tubes (Horne et al., 2009; Shao et al., 2011). Therefore, a higher estrogen concentration during the assisted reproduction cycle may alter the normal physiological function of the fallopian tube, which increases the incidence of tubal pregnancy (Shao et al., 2012).

### 7.3 Preeclampsia

Preeclampsia affects 3%–5% of all pregnancies and is a severe life-threatening pregnancy-related disorder and the leading cause of neonatal mortality and morbidity (Vigil-De Gracia, 2009). This systemic hypertensive disorder has different phenotypes that are triggered by distinct underlying mechanisms occurring at the early stage of pregnancy (Than et al., 2018). The key signature of preeclampsia is presented by models showing inadequate spiral artery invasion precluding normal placentation (Maltepe et al., 2010; Hunkapiller et al., 2011). Studies using mouse models have revealed that Notch signaling activity is highest in eEVTs and that the conditional depletion of *Notch2* reduces eEVT arterial invasion and placental perfusion, leading to embryo lethality (Hunkapiller et al., 2011). Furthermore, studies performed using placental tissues obtained from patients with preeclampsia showed that *Notch* ligand expression is absent in EVTs located at perivascular and endovascular areas (Hunkapiller et al., 2011). In addition to arterial invasion, poor trophoblast migration and endothelial remodeling are other events associated with preeclampsia (Lam et al., 2005). Evidence from many studies (including ours) has shown that endothelial dysfunction is the primary cause of clinical features in patients with preeclampsia (Roberts and Cooper, 2001; Yi et al., 2021). In particular, endothelial dysfunction leading to a decrease in vascular tone causes hypertension, increased glomerular vascular permeability causes proteinuria, and decreased coagulation factor causes coagulopathy (Lam et al., 2005). Additionally, subsequent endothelial injury induces vasoconstriction and ischemia and gives rise to liver dysfunction (Lam et al., 2005). Given that preeclampsia occurs more often in first pregnancies, it is most likely that an increase in reproductive hormones and pregnancy-related factors during the first pregnancy prime the reproductive system for subsequent pregnancies (Cha et al., 2012).

### 7.4 Preterm labor

Preterm labor (also known as premature birth) is defined as labor that begins before 37 weeks, and it accounts for 75% of early neonatal morbidity and mortality (Goldenberg et al., 2008). Studies performed using a mouse model have demonstrated that preterm labor can adversely result from defective decidualization at the maternal site (Hirota et al., 2010). Transformation-related protein 53 (Trp53), which encodes p53, is a tumor suppressor gene that also plays a critical role in regulating female reproduction (Vogelstein et al., 2000; Brosens and Gellersen, 2006). Constitutive depletion of *Typ53* in mice results in implantation failure due to the downregulation of LIF on E4 (Hu et al., 2007). Conditional uterine depletion of Trp53 in mice results in normal implantation, whereas most of these mice have an increased incidence of preterm birth, which can be corrected by administering a selective cyclooxygenase-2 (COX-2) inhibitor (Hirota et al., 2010). Notably, these mutant mice exhibit compromised decidualization accompanied by an increase in terminally differentiated decidual cells with polyploidy, indicating a regulatory role for Trp53-LIF-COX-2 signaling in uterine decidualization and placentation stabilization (Hirota et al., 2010). The mammalian target of rapamycin complex 1 (mTORC1) signaling is an important molecular mechanism that triggers cellular senescence, and rapamycin (an mTORC1 inhibitor) attenuates senescence, which increases the life span of mice (Harrison et al., 2009). Intriguingly, subsequent studies have indicated that the decidua of Trp53 mutant mice has increased the mTORC1 activity that can be suppressed by administering rapamycin, leading to the rescue of preterm birth because of the attenuation of premature decidual senescence (Hirota et al., 2011). Consistent with these animal studies, clinical information shows that pregnant women of advanced age have an increased risk of preterm labor (Cnattingius et al., 1992; Nelson and Lawlor, 2011).

## 8 Clinical applications and therapeutic potential

### 8.1 Maternal-fetal cellular trafficking

Maternal-fetal cellular trafficking is the bidirectional passage of cells between a mother and her fetus during pregnancy, resulting in the presence of fetal cells in the maternal circulation (a phenomenon called fetal microchimerism) as well as the presence of maternal cells in the fetal circulation (maternal microchimerism) (Bianchi et al., 1996; Maloney et al., 1999). This cellular trafficking begins at 7 weeks of gestation and steadily increases throughout gestation, with a peak level at parturition (Ariga et al., 2001). The underlying mechanisms of this physiological phenomenon involve vascular endothelial growth factor- and integrin-dependent signaling pathways and HLA class II molecules; however, the factor that triggers these signaling pathways remains unclear (Chen et al., 2008; Hahn et al., 2019). Studies have shown that alterations in maternal-fetal cellular trafficking are associated with the disruption of the fetal-maternal interface due to preeclampsia, termination of pregnancy, and fetal surgery, suggesting a regulatory role for the placenta in cell trafficking (Holzgreve et al., 1998; Bianchi et al., 2001; Saadai et al., 2012; Hahn et al., 2019).

Nonshared HLA-DR alleles (informative alleles) between the fetus and mother are used to detect and distinguish maternal-fetal microchimerism in human blood and tissues (Nijagal et al., 2011). This bidirectional passage of cells has been widely implicated in various pathophysiological conditions, including maternal immune tolerance, the fetal immune system, immune surveillance, tissue repair in autoimmune diseases and cancers, and the delicate balance between immunological priming and tolerance in organ transplantation (Jeanty et al., 2014). The related technique has been clinically applied for the prenatal diagnosis of fetal aneuploidy and the prediction of pregnancy complications, such as preeclampsia and preterm labor (Farina et al., 2004; Jakobsen et al., 2012). Ongoing studies have shown that the placenta is the major source of cell-free fetal nucleic acids in maternal circulation (Faas et al., 2012; Tsang et al., 2017). Given that levels of cell-free DNA and placenta-specific RNA transcripts are elevated in the maternal circulation of women with preeclampsia, preterm labor, and restricted fetal growth, detecting these cell-free fetal nucleic acids can be a useful, noninvasive tool for placental functional monitoring (Leung et al., 1998; Pang et al., 2009; Zhang et al., 2016). During trophoblast invasion and throughout pregnancy, the discovery of extracellular vesicles (EVs) has deepened our understanding of immune modulation as local or systemic carriers of antigens and immune-regulatory molecules (Morelli and Sadovsky, 2022). Novel findings of immune-regulatory molecules located on EVs or within their cargo indicate that EVs play an essential role in exerting immune tolerance during human pregnancy. Recent findings from transplant immunology studies suggest molecular interactions between fetus- or placenta-derived EVs and maternal lymphoid tissues (Morelli and Sadovsky, 2022).

### 8.2 Placenta-enriched molecules

The placenta is a temporary organ of fetal origin and thus has a unique transcriptome and proteome. Indeed, a number of miRNAs, mRNAs, and proteins are either exclusively or highly expressed in the placenta compared to other human tissues. These placenta-enriched molecules have been identified and are detectable in the maternal peripheral blood, and they decay rapidly after delivery. The measurement of these unique molecules has been considered for use as potential biomarkers for pregnancy-related disorders (Whigham et al., 2019). Recent studies have focused on targeting several placenta-enriched molecules as a therapeutic strategy for placental dysfunction and pregnancy complications. In the human genome, approximately 40% of miRNAs are present in clusters, some of which are placenta-specific or primarily expressed in the placenta (Altuvia et al., 2005). The C19MC cluster is a primate-specific miRNA cluster that is exclusively inherited in the placenta and derived from the paternal allele (Noguer-Dance et al., 2010). C14MC is another placenta-specific miRNA cluster inherited from maternally imprinted genes (Seitz et al., 2004). Notably, the circulating levels of miRNA from the C19MC cluster steadily increase throughout pregnancy, whereas the circulating levels of miRNA from the C14MC cluster decrease throughout pregnancy (Morales-Prieto et al., 2012). The differential expression patterns of these two placental miRNA clusters may indicate their regulatory roles at different gestational stages. Several placental miRNAs are derived from primary trophoblasts and are linked to hypoxia (Mouillet et al., 2010; Lee et al., 2011). These hypoxia-related miRNAs are measurable in the maternal peripheral blood throughout pregnancy as the fetus encounters progressive placentation-induced hypoxia, suggesting a potential assessment tool for fetal health and placental function (Whitehead et al., 2013a).

Similarly, several placenta-specific mRNA transcripts were detected in the maternal peripheral blood that rapidly disappeared after delivery (Ng et al., 2003; Whigham et al., 2019). Some unique placental mRNA transcripts have been identified as being differentially expressed in pathological conditions representing placental dysfunction (Whigham et al., 2019). For instance, the mRNAs of *CRH*, *PLAC3*, *PLAC4*, and *ERVWE1* are significantly increased in maternal plasma and placental tissue obtained from women with preeclampsia (Paiva et al., 2011). Additionally, the levels of some placental mRNA transcripts, such as adrenomedullin, are positively correlated with increased fetal hypoxia and fetal growth restriction during the progression of gestation, which is also highly associated with abnormal findings in fetal vessels examined using Doppler velocimetry (Whitehead et al., 2013b). These findings suggest that these placental mRNA transcripts are promising biomarkers for assessing the health of both the fetus and the placenta. In addition to miRNAs and mRNA transcripts, placenta-enriched proteins have long been studied to predict and prevent placental dysfunction and to improve maternal and fetal surveillance. Among these placenta-enriched proteins, hCG is the most popularly applied biomarker. At approximately 2 weeks after implantation, this biomarker is detectable in urine and blood samples to determine the pregnancy status and further differentiate normal and ectopic pregnancies (Murray et al., 2005). Combined with ultrasonographic markers, maternal circulating hCG concentrations are commonly used as a prenatal screen test for fetal Down syndrome (Trisomy 21) during the first trimester (Wald et al., 1996). Pregnancy-associated plasma protein A (PAPP-A) is exclusively expressed by trophoblasts in the placenta and is involved in placental development and fetal growth (Bolnick et al., 2016). During the first trimester, several circulating placenta-derived proteins have been compared in prenatally predicting fetuses that are small-for-gestational-age (SGA, body weight <10th centile), and PAPP-A is the most reliable biomarker (Zhong et al., 2015). Placental growth factor (PlGF) is a promising placenta-enriched protein that is produced by STs. This placental protein is an angiogenic factor that has been demonstrated to be diminished in the serum of women with preeclampsia and gestational diabetes mellitus (Reuvekamp et al., 1999; Carmeliet et al., 2001; Yanachkova et al., 2023). As early as 13–16 weeks of gestation, pregnant women who subsequently develop preeclampsia have lower serum levels of PIGF than the controls, highlighting a potential biomarker for predicting the early onset of preeclampsia (Levine et al., 2004). Intriguingly, this placental protein has also recently been reported to predict infants born with SGA (Gaccioli et al., 2018). These findings suggest that PIGF is most likely an indicator of overall placental function rather than a marker of a specific placental disease (Whigham et al., 2019).

Soluble fms-like tyrosine kinase 1 (sFlt-1) is a natural slice variant of the vascular endothelial growth factor-A (VEGFA) receptor Flt-1 lacking the transmembrane and cytoplasmic domains and it acts as a potent antagonist against VEGFA and PlGF (Kendall et al., 1996). Many studies have demonstrated that the maternal circulating levels of sFlt-1 are significantly elevated in women with preeclampsia (Maynard et al., 2003; Whigham et al., 2019). Studies performed using preeclampsia placental tissues have shown that the expression of VEGFA is upregulated in maternal decidual cells, whereas the expression of sFlt-1 is highly overexpressed in fetal EVTs that invade the decidua, indicating that EVTs overexpress sFlt-1 in self-defense against excessive VEGFA production by maternal decidual cells (Fan et al., 2014; Whigham et al., 2019). Therefore, the sFlt-1/PlGF ratio has been clinically applied as a predictive value in women with suspected preeclampsia (Zeisler et al., 2016). Information obtained from clinical studies shows that the circulation levels of sFlt-1 are directly proportional to the severity of preeclampsia (Chaiworapongsa et al., 2004; Thadhani et al., 2004). Specifically, sFlt-1 concentrations are relatively higher in women with preeclampsia of more severe grades, in earlier onset of disease, and with SGA infants (Lam et al., 2005; Modzelewski et al., 2023). Clinical studies suggest that diagnostic indicators using the circulating sFlt-1/PlGF ratio in combination with ultrasonographic parameters increase the predictive value for severe fetal growth restriction (Gaccioli et al., 2018).

### 8.3 Therapeutic strategies targeting placenta-enriched molecules

To date, effective therapeutic options for pregnancy diseases (especially preeclampsia and fetal growth restriction) are limited, even though many potential therapeutic strategies have been proposed (Onda et al., 2017; Pepe and Albrecht, 2021). Because many drugs are small molecules that can pass the placenta to enter the fetal body, it has been challenging to make reformulated medications with potentially detrimental effects on the fetuses. Upcoming clinical studies performed using specific targeted designs for specific placenta-enriched molecules seem to be a reliable strategy for prenatal medicine. These methods are designed to reduce the dose of drugs and enhance delivery efficiency, thus minimizing fetal exposure to the medications. At present, several treatment strategies targeting placenta-enriched molecules have emerged.

Epidermal growth factor receptor (EGFR) is a transmembrane glycoprotein that is highly expressed in the placenta compared to other noncancerous human tissues, indicating that EGFR is also a placenta-enriched protein (Wu et al., 2009). A phase II clinical trial proposed a combination therapy using methotrexate and the EGFR inhibitor gefitinib to treat patients with ectopic pregnancies (Whigham et al., 2019). Nanoparticle-targeted drug therapy is designed to achieve controlled drug release and disease-specific localization by optimizing the polymer characteristics (for reviews, see (Singh and Lillard, 2009; Leziak et al., 2022)). This nanoparticle-based technique has been exploited to pack doxorubicin (a chemotherapeutic reagent) into drug-delivery vehicles incorporated with antibodies against EGFR, which are delivered into the trophoblasts to treat patients with ectopic pregnancies (Kaitu’u-Lino et al., 2013). In animal studies performed using the same strategy, methotrexate (a chemotherapeutic reagent) is packaged into nanoparticles incorporated with a specific placental marker and delivered into the mouse placenta to impair the development of the placenta and fetus in mice dramatically (Zhang et al., 2018).

In addition to its diagnostic potential for preeclampsia, sFlt-1 has been a considerable focus of research regarding therapeutic approaches. It is proposed that the targeted reduction in the circulating levels of sFlt-1 will be of clinical benefit in controlling disease progression and prolonging pregnancy duration for women with preeclampsia (Rduch et al., 2023). Several studies have shown a reduction in the placental secretion of sFlt-a1 following the administration of several small-molecule inhibitors, including pravastatin, esomeprazole, and metformin (Brownfoot et al., 2015; Brownfoot et al., 2016; Onda et al., 2017). A study using a small interfering RNA (siRNA)-mediated approach demonstrated successful selective silencing of sFlt-1 mRNA transcripts in mouse placenta (Turanov et al., 2018). Animal studies performed by the same study group using a baboon preeclampsia model have demonstrated that a single dose of siRNA suppresses the overexpression of sFlt-1 and relieves the clinical signs of preeclampsia (Turanov et al., 2018). These findings suggest that treatment using RNA interference that targets placenta-enriched molecules could be a novel therapeutic strategy for pregnancy dysfunction.

## 9 Conclusion

The establishment of the fetal-maternal interface predominantly relies on highly organized events, including blastocyst development, implantation, decidualization, trophoblast differentiation and invasion, and placentation. The success of each event involves an intricate succession of various genetic and cellular interactions that must be executed in an appropriate manner and within an optimal time frame. A better understanding of these fundamental events is imperative to explain the underlying molecular mechanisms and pathogenesis of failed implantation, inadequate placentation, placental dysfunction, and pregnancy-related diseases. Although many functions of this adaptable organ have yet to be uncovered, increasing evidence suggests that placental health has a dramatic impact on the short- and long-term consequences of the developing fetus. The development of scRNA-seq and the derivation of human TS cells and the CTB 3D-organoid culture system has created novel tools to explore the delicate and complicated niche built by the crosstalk between trophoblasts and the decidual endometrium. Analyses of the placenta-specific transcriptome and proteome have identified several unique gene products in the maternal circulation that are clinically applied as potential noninvasive biomarkers of placental dysfunction and diseases. The clinical potential of appropriately designed therapeutic strategies targeting placenta-enriched molecules in various pregnancy dysfunctions and diseases has been demonstrated recently. Although some medical agents are in clinical trials, practical issues related to bioavailability and safety must be critically evaluated.
